# Is salamander arboreality limited by broad-scale climatic conditions?

**DOI:** 10.1371/journal.pone.0255393

**Published:** 2021-08-18

**Authors:** Erica K. Baken, Lauren E. Mellenthin, Dean C. Adams

**Affiliations:** 1 Department of Science, Chatham University, Pittsburgh, Pennsylvania, United States of America; 2 Department of Ecology and Evolutionary Biology, Yale University, New Haven, Connecticut, United States of America; 3 Department of Ecology, Evolution, and Organismal Biology, Iowa State University, Ames, Iowa, United States of America; Instituto Federal de Educacao Ciencia e Tecnologia Goiano - Campus Urutai, BRAZIL

## Abstract

Identifying the historical processes that drive microhabitat transitions across deep time is of great interest to evolutionary biologists. Morphological variation can often reveal such mechanisms, but in clades with high microhabitat diversity and no concomitant morphological specialization, the factors influencing animal transitions across microhabitats are more difficult to identify. Lungless salamanders (family: Plethodontidae) have transitioned into and out of the arboreal microhabitat many times throughout their evolutionary history without substantial morphological specialization. In this study, we explore the relationship between microhabitat use and broad-scale climatic patterns across species’ ranges to test the role of climate in determining the availability of the arboreal microhabitat. Using phylogenetic comparative methods, we reveal that arboreal species live in warmer, lower elevation regions than terrestrial species. We also employ ecological niche modeling as a complementary approach, quantifying species-level pairwise comparisons of niche overlap. The results of this approach demonstrate that arboreal species on average display more niche overlap with other arboreal species than with terrestrial species after accounting for non-independence of niche model pairs caused by geographic and phylogenetic distances. Our results suggest that occupation of the arboreal microhabitat by salamanders may only be possible in sufficiently warm, low elevation conditions. More broadly, this study indicates that the impact of micro-environmental conditions on temporary microhabitat use, as demonstrated by small-scale ecological studies, may scale up dramatically to shape macroevolutionary patterns.

## Introduction

How and why organisms transition to, and then occupy previously uninhabited microhabitats is of major interest to biologists. A variety of ecological and evolutionary patterns have been identified as mechanisms that promote or support transitions across microhabitats. For example, the Caribbean *Anolis* lizards repeatedly colonizing specific arboreal microhabitats has been attributed to interspecific competition [1]. Fossil evidence of post-cranial skeletal adaptations in early tetrapods demonstrate the importance of morphological adaptation in promoting the vertebrate transition to life on land [2], and genome duplication has allowed many plant lineages to colonize mountain tops and other previously uninhabited regions [3]. Arboreal salamanders represent a similarly intriguing habitat transition; salamanders’ strong dependence on water for nearly all aspects of life makes tree-tops an unlikely habitat for these creatures. Despite this, salamanders have transitioned to arboreal life several times across the history of the order [4], yet the mechanisms behind these transitions remain largely unexplained.

Across salamanders’ (order: Caudata) evolutionary history, arboreality has evolved at least five times and has been subsequently lost nearly 60 times [4]. Baken and Adams [4] also found a dramatic difference in transition rates between terrestrial and arboreal life, such that species abandon arboreal microhabitats 23 times more readily than they colonize them. These discoveries suggest that some strong force is limiting the availability of the arboreal microhabitat on a macroevolutionary scale, yet an explanation for the macroevolutionary dynamics and the present state of salamander arboreality remains elusive. For salamanders, arboreal life does not require morphological specialization of body or foot shape [4, 5], indicating that the occupation of arboreal microhabitats is not constrained by locomotive challenges. It is also unlikely that arboreality is maintained primarily by competition among species, as arboreal salamanders are found both in sympatry and allopatry with terrestrial salamanders [6]. One remaining, and as of yet unexplored, possible explanation is that climatic conditions dictate whether the arboreal microhabitat is suitable for salamanders.

For this study, we suggest that abiotic conditions may act as a determinant of primary microhabitat use in lungless salamanders for several reasons. First, it is well-established that local environmental conditions influence plethodontid landscape movement and short-term microhabitat selection [7–12]. Second, Baken and colleagues [13] revealed a strong correlation between broad climatic patterns and plethodontid surface area to volume ratio (SA:V) evolution, demonstrating the power of climate to influence macroevolutionary patterns within this family. Finally, SA:V, the trait shown to be tied to climatic variation, is directly related to two physiological demands that are amplified by arboreal life: moisture conservation and oxygen acquisition. Arboreal microhabitats inherently impose a higher risk of desiccation than do nearby terrestrial microhabitats, as they lack the cover objects (e.g., leaf litter, fallen logs), humidity, and protective boundary layer of the forest floor (although arboreal refugia exist, such as pools of rainwater caught by arboreal bromeliads [14]). Lizards also use greater energetic resources when climbing around the complex, three-dimensional environment of a tree [15, 16], and we propose this energetic cost might also be present for salamanders (although the precise costs might depend on body size). Consolidating these lines of evidence, we hypothesized that the arboreal microhabitat is only available to plethodontids under certain climatic conditions that promote humidity and efficient cutaneous oxygen exchange. Thus, we predicted that arboreal species occupy geographic ranges with climates characterized by high humidity and low elevation (to accommodate an increased demand for oxygen available at lower elevations), whereas terrestrial species can occupy a wider variety of climates.

To address our hypothesis, we extracted and summarized present-day climatic variables from new-world plethodontid species’ occurrence data. We then conducted a series of multivariate phylogenetic ANOVAs to compare the climatic conditions between species’ use of arboreal and terrestrial microhabitats. While these methods can appropriately account for phylogenetic relatedness, they require that data points across species have the same dimensionality, necessitating the reduction of climate data to summary statistics. Thus, we sought to complement our main analyses with ecological niche modeling, which does not require such summarization. For this ecological niche modeling approach, we quantified niche overlap across each species pair, and compared intra-microhabitat niche overlap to inter-microhabitat niche overlap while accounting for geographic and phylogenetic distances. Our results from both approaches indicate that salamander arboreality is only observed in regions characterized by warm temperature, high potential evapotranspiration, and low elevation, implicating climate as an influential factor in determining microhabitat use across Plethodontidae.

## Materials and methods

The family of lungless salamanders, Plethodontidae, makes up roughly 65% of all extant salamander species (28 genera, ~478 out of 738 named species [17]), is geographically widespread, and includes all known extant arboreal salamanders [4]. Although recent physiological work has demonstrated that salamanders display greater resistance to water loss than previously considered [see 18, 19], their skin still has relatively less resistance to evaporative water loss as compared to other vertebrate taxa [20]. Thus they are relatively more prone to desiccation, and as a consequence most species are often observed utilizing rocks and logs to hide under for desiccation avoidance [14]. Adult body sizes range from 15 mm to over 100 mm snout-vent length [4], and species occupy a wide variety of microhabitat types, including terrestrial, aquatic, and arboreal microhabitats [4, 5, 14]. Most plethodontid species live in North America; however, several species live in South America, Europe, and South Korea [14, 17]. For this study, we focused on 277 North and South American plethodontids for which appropriate occurrence, phylogenetic, and microhabitat data were available.

### Microhabitat use

We classified species’ primary and, where applicable, secondary adult microhabitat based on primary literature, species descriptions, field observations, and the IUCN [21] and AmphibiaWeb [17] databases. From these primary and secondary microhabitat categories, we applied six variable classification schemes following [4]. This allowed us to account for the non-discrete nature of microhabitat use in this family as well as the possible importance of secondary microhabitats in driving ecological and evolutionary patterns. Briefly, species were assigned a microhabitat type by translating their primary and secondary microhabitats into a single category (terrestrial: T, arboreal: A, cave-dwelling: C, fossorial: F, saxicolous: S, aquatic: W, or semi-aquatic: SW) according to three considerations: whether a separate category of ‘semi-aquatic’ (SW) species was included, the data source defining arboreality, and the inclusion of secondary microhabitats (majority-rule, ‘-M’, or lenient rules, ‘-L’; Table 1).

**Table 1:** Microhabitat classifications schemes.

Scheme	Majority Rule or Lenient	Possible Microhabitat Categories	Alternative Arboreality Data Source
*6-M*	Majority Rule	A, C, F, S, T, W	*NA*
*6-L*	Lenient	A, C, F, S, T, W	*NA*
*7-M*	Majority Rule	A, C, F, S, T, W, SW	*NA*
*7-L*	Lenient	A, C, F, S, T, W, SW	*NA*
*6-McM*	Majority Rule	A, C, F, S, T, W	McEntire 2016
*6-McL*	Lenient	A, C, F, S, T, W	McEntire 2016

This resulted in six different classification schemes (6-M, 6-L, 7-M, 7-L, 6-McM, and 6-McL), where the schemes with ‘7-’ as their first designator included semi-aquatic as a separate species (semi-aquatic species were classified as either aquatic or another non-aquatic microhabitat type under the other schemes). Schemes with ‘-Mc-’ utilized an independent data source for classifying species as arboreal (McEntire’s species classifications of arboreality, which includes vegetation-climbers as arboreal [22]). This last consideration of majority-rule (‘-M’) versus lenient (‘-L’) schemes differed in that majority-rule schemes simply reflected the primary microhabitat, whereas the lenient schemes considered a species to be non-terrestrial if either their primary or secondary microhabitat was non-terrestrial. As most results were consistent across classification schemes, we reported the 6-M results in the main text, indicated when alternative classification schemes contradicted 6-M results, and results from all classification schemes can be found in the Supporting information. Although this study focuses on arboreal and terrestrial species, other species were included in the analysis to accurately encapsulate the full range of statistical climatic variation inhabited by this family.

The ‘Majority Rule or Lenient’ column defines which schemes used species’ secondary microhabitats to define microhabitat use (applied when primary microhabitat was terrestrial). In these schemes, species were categorized to describe their more specialized microhabitat under the assumption that the terrestrial microhabitat is the more generalized microhabitat. The column ‘Possible Microhabitat Categories’ distinguishes between schemes that considered ‘semi-aquatic’ to be a category distinct from ‘terrestrial’ or ‘aquatic’, as has been done in other related papers. The final column identifies the schemes for which an alternative data source for defining arboreality was employed. This source [22] only listed facultative and obligately arboreal species, so for species not mentioned in that manuscript, classifications matched the other schemes with the corresponding criteria. Detailed description of each classification scheme can be found in [4], and species’ classifications under each scheme are provided in the S1 Table.

### Distribution and environmental data

As described below, environmental patterns were evaluated using two complementary approaches: ecological niche modeling (based on all locality data), and phylogenetic comparative analyses (based on species-level mean data). For both approaches, we first obtained environmental parameters using species occurrence data for 277 North and South American plethodontid species from the online VertNet (accessed 8/17/2020 [6]) and Global Biodiversity Information Facility (accessed 10/27/2020, www.gbif.org) databases. These datasets were pruned such that outliers and erroneous points were excluded, erring on the side of removing questionable points. Multiple occurrence points within the same climate pixel (described below) were also excluded to avoid issues of spatial autocorrelation. For the species represented by at least three occurrence points (after spatial autocorrelation pruning), we then extracted 12 climate variables from each verified occurrence point, resulting in climate data from 56,092 localities (3 to 7358 localities per species, mean = 202.5). Climatic variable selection was limited to those publicly available across the full geographic extent occupied by the included species and followed previous studies of amphibian niche variation [23–25] and microhabitat use in plethodontids [11, 26–29] to capture abiotic factors that are known to influence plethodontid survival and behavior. These variables represent yearly averages and extremes of climate variation collected over the past 40 years and included nine WorldClim variables for air temperature, precipitation, and potential evapotranspiration (Annual Mean Temperature: BIO1, Maximum Temperature of the Warmest Month: BIO5, Minimum Temperature of the Coldest Month: BIO6, Annual Precipitation: BIO12, Precipitation of the Wettest Quarter: BIO16, Precipitation of the Driest Quarter: BIO17: [30]; Annual PET: PET.A, PET of the Wettest Quarter: PET.W, and PET of the Driest Quarter: PET.D: [31]). We also included elevation (Elev: [31]), yearly climatic moisture (CM: [31]), and yearly average cloud cover (CC) following Peterson and Nakazawa [32] (IPCC 2001, 0.5 arc minute resolution resampled to 2.5 arc minute resolution to match the other climate datasets, ~4.5 km at equator). Other climate variables, such as Water Vapor Pressure, were excluded due to autocorrelation with other variables or exclusion in other related investigations. We acknowledge that climatic data obtained in this fashion are coarse-grained, and that intraspecific variation in both activity patterns and microhabitat use may obscure the association between microhabitat use and the climatic environment. However, recent work on plethodontid climatic envelopes, obtained at both micro- and macro-scales reveals a close association between the two [12], implying that the climate variables extracted for this study may serve as a first approximation for describing the general patterns and boundaries of microenvironments available to individual salamanders (see Discussion).

### Phylogenetic comparative analyses

We used a phylogenetic comparative approach to assess differences in climatic patterns between microhabitat types. Here, species-specific summary statistics (i.e., multivariate species means) were obtained for each climate variable, resulting in five values per climate variable (5th percentile, 25th percentile, mean, 75th percentile, and 95th percentile) per species (S1 Table). We then performed several phylogenetic ANOVAs of climate and microhabitat, examining pairwise comparisons of arboreal versus terrestrial species. We assessed significance via randomized residual permutation procedures [33, 34] in the package, RRPP [35, 36] using R v.3.6.0 [37] and accounted for multiple comparison error rate inflation via sequential Bonferroni.

The first phylogenetic ANOVA tested whether the combined dataset of all summary statistics differed significantly between arboreal and terrestrial species. As the raw variables were represented in incommensurate units, the summary statistics were converted to standard normal deviates (μ = 0; std = 1) to allow for meaningful multivariate analysis [see 38, 39]. Because climate variables are often highly correlated, we next performed a principal component analysis (PCA) on the normalized data and extracted the first five PC axes (explaining >87% of the overall climate variation) to test against microhabitat. Finally, we tested each climate variable separately (each climate variable was a five-dimensional variable of the non-normalized summary statistics).

To account for similarity due to evolutionary relatedness, all phylogenetic ANOVAs used the time-calibrated phylogeny from [40] of 516 Caudata species. This phylogenetic reconstruction was based on three mitochondrial and four nuclear genes and used Bayesian approaches under a pure-birth speciation prior on the tree topology and divergence times, an uncorrelated lognormal molecular clock, and 12 node calibrations from Shen et al. ([41]; for additional details see [40]). We pruned the maximum clade credibility tree to match the species used in this study.

To test the robustness of our results, we repeated all analyses across each microhabitat classification scheme described above and on a reduced dataset of only tropical arboreal and tropical terrestrial species under each classification scheme (132 species under 6-M). This tropical robustness consideration addresses the potential bias caused by the unequal representation of arboreality across temperate and tropical regions (arboreality is more common in tropical clades). We also calculated uncertainty intervals of all Z statistics by repeating the analyses across the 1000 pruned posterior chronograms from [40] phylogenic reconstruction to account for phylogenetic uncertainty.

### Ecological niche modeling

Next, we used ecological niche modeling (ENM) to complement our main phylogenetic comparative analyses. Specifically, we constructed niche models for each species, based on climatic data from all geographic localities, using the maximum entropy algorithm implemented in MAXENT v3.4.1 [42, 43] using the R package, dismo [44]. This algorithm generally outperforms other ENM methods [43, 45, 46] and is less sensitive to sample size [47]. For this analysis, we only included species with 15 or more occurrence points available after pruning (183 species, 15 to 7358 localities per species, mean = 302.5), as MAXENT demonstrates strong predictive abilities with as few as 15 localities [48]. We then quantified degree of niche overlap between each species pair using two common metrics, Schoener’s *D* and Warren’s *I* [49] (‘nicheEquivalency’ function in the R package dismo; [44]). These similarity metrics range from 0 (no niche overlap) to 1 (identical niches) and are calculated by taking the difference between projected suitability scores across microhabitat models within each map pixel.

We then evaluated the mean value of niche overlap for three types of microhabitat comparisons: inter-microhabitat pairs of one arboreal and one terrestrial species (AT pairs), and intra-microhabitat pairs of either two arboreal species (AA pairs) or two terrestrial species (TT pairs) as defined by the various microhabitat classification schemes. To account for non-independence due to geographic proximity and phylogenetic history between species, we first regressed the niche overlap values onto both geographic and phylogenetic distances of the species pairs. Geographic distance was quantified as the Great Circle (WGS84 ellipsoid) distance between mean occurrence points for each species using the ‘spDists’ function in the sp package in R [50, 51], and phylogenetic distance was extracted from the phylogenetic variance-covariance matrix constructed using the ‘vcv’ function in the ape package in R [52]. We then extracted the residuals from that regression to test against microhabitat pairing type (AA, TT, and AT) in an ANOVA framework. Significance was assessed using residual randomization procedures in a similar manner as the comparative approach described above. These ENM analyses were repeated across all microhabitat classifications and across the 1000 pruned posterior chronograms as above.

## Results

### Phylogenetic comparative analyses

The results from all phylogenetic ANOVAs across microhabitat schemes of the full species dataset were in agreement, thus only results from the 6-M analysis are presented below with exceptions noted. 6-M was selected here because it represents the most conservative distinction between arboreal and terrestrial species; it only classifies species as arboreal when they spend the majority of their lives in trees (in contrast to all ‘-L’ schemes), it does not distinguish semi-aquatic species from terrestrial species that occasionally occupy aquatic microhabitats (in contrast to ‘7-‘ schemes), and species that climb tall leafy vegetation, but not explicitly trees, are not considered arboreal (in contrast to’ -Mc-‘ schemes). Results from all analyses are available in the Supporting information.

**Fig 1:**
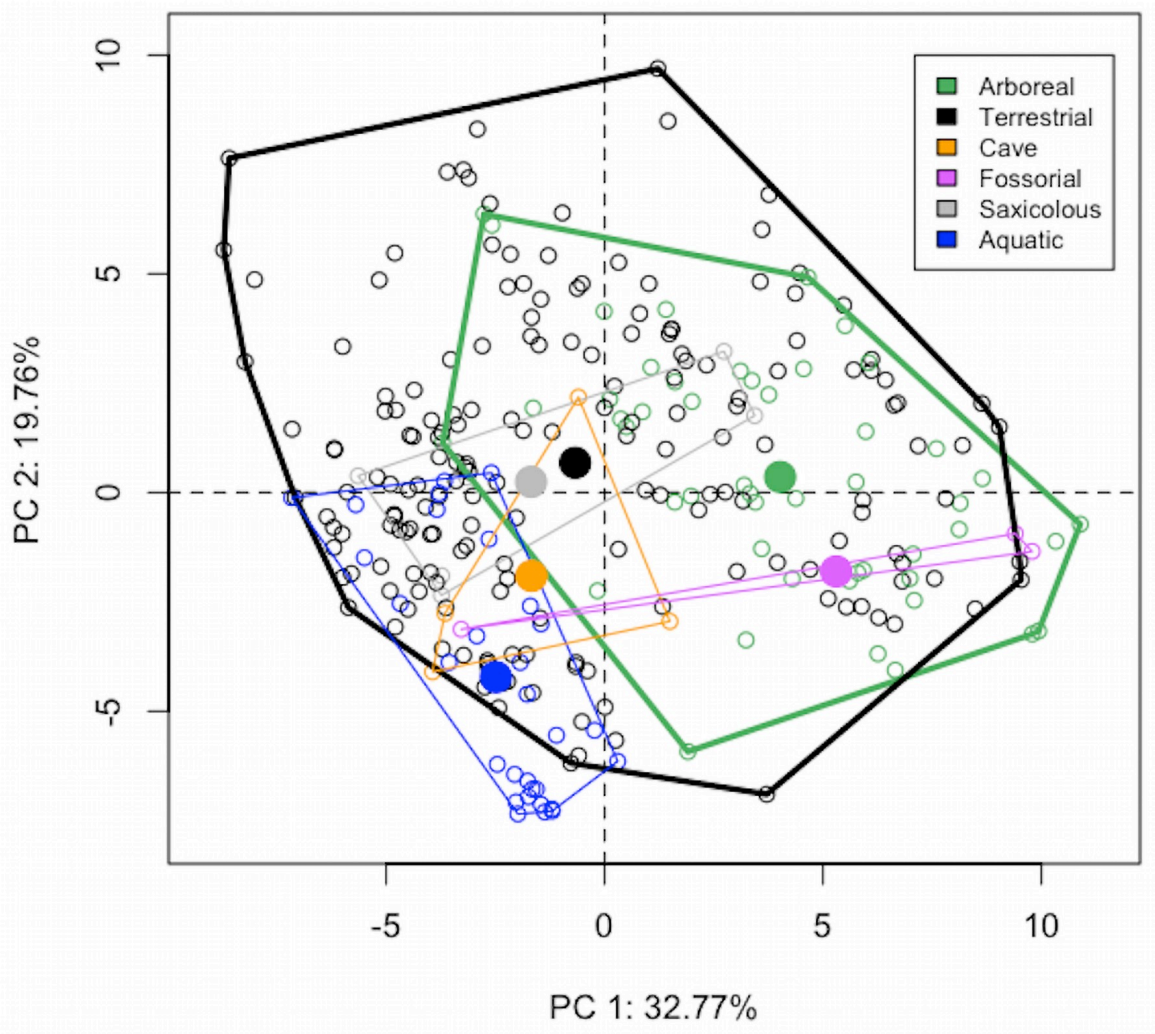
PCA of climate variables. All species are colored by microhabitat type under the 6-M classification scheme. Small, unfilled points indicate species’ means, and larger solid points indicate microhabitat-wide means. Polygons encapsulating all species within a microhabitat group are also included. Arboreal species on average display higher PC1 values, which corresponds to warmer temperatures, more precipitation, and lower elevations (see S2 Table for full loading data). This plot also suggests that although the means differ between arboreal and terrestrial species, these groups do not occupy fully distinct climate spaces. Rather, arboreal species appear to occupy a subset of the climates occupied by the terrestrial species.

As predicted, the phylogenetic ANOVA assessing all climate variables together revealed significant differences in group mean climates across microhabitat types (Z = 2.326, p = 0.011; Fig 1) and between arboreal and terrestrial species (pairwise comparison: Z = 5.025, p = 0.001; Table 2). We found similar patterns in the pairwise comparison analyses of the first two PC axes (Z_PC1_ = 4.644, p_PC1_ = 0.001; Z_PC2_ = 4.198, p_PC2_ = 0.002; Table 2 and Fig 1), with arboreal species on average displaying higher PC1 values (Δgls-coefficient = 1.362) and lower PC2 values (Δgls-coefficient = -1.339) than terrestrial species after accounting for phylogeny. PC1 represented 32.77% of the overall variation (Table 3) and was most heavily driven by multiple or all summary statistics for Annual Mean Temperature (BIO1), Min Temperature of the Coldest Month (BIO6), Annual Precipitation (BIO12), and Precipitation of the Wettest Quarter (BIO16; all loaded positively; S2 Table), implying high PC1 values represent climates with warmer cold seasons, more extreme wet seasons. PC2 represented 19.76% of the overall variation (Table 3) and was driven by all summary statistics for Max Temperature of the Warmest Month (BIO5; negative loading) and Elevation (positive loading), along with some summary statistics for Potential Evapotranspiration of the Wettest Quarter (PET.W, negative loading; S2 Table) such that low values of PC2 represent low elevation, humid regions with hot summers. PC axes 3–5 did not show significant differences between arboreal and terrestrial species. These results were consistent across all classification schemes except 6-McL, where despite a significant difference in overall climate (Z = 2.651, p = 0.010), PC1 was only marginally significant (Z = 2.219, p = 0.038, sequential Bonferroni-adjusted α = 0.010), and PC2 was not significant (Z = 1.280, p = 0.127; S3 Table; see Discussion). Further, in testing phylogenetic uncertainty using the 1000 posterior chronograms, the lower bound of the confidence interval around the overall climate Z-score under 6-McL overlaps with the significance cutoff of 1.645 (S1 Fig).

**Table 2:** Pairwise Z scores and p values from all phylogenetic ANOVAs.

	Variable	Z	P	Alpha
	All	5.025	0.001	0.0500
	PC1	4.644	0.001	0.0100
	PC2	4.198	0.002	0.0125
	PC3	-0.547	0.656	0.0500
	PC4	2.550	0.022	0.0167
	PC5	1.559	0.088	0.0250
Temperature	BIO1 (Annual)	5.941	0.001	0.0042
	BIO6 (Cold)	6.311	0.001	0.0045
	BIO5 (Warm)	4.365	0.003	0.0056
Precipitation	BIO12 (Annual)	1.270	0.116	0.0100
	BIO16 (Wet)	1.385	0.103	0.0083
	BIO17 (Dry)	-0.394	0.588	0.0167
	PET.A	3.281	0.010	0.0063
	PET.W	-0.332	0.564	0.0125
	PET.D	1.392	0.102	0.0071
	CM	-0.509	0.654	0.0250
	Elevation	7.280	0.001	0.0050
	Cloud	-0.749	0.775	0.0500

These results were generated from the full dataset under the 6-M classification scheme. Also displayed are the appropriate sequential-Bonferroni adjusted alpha values against which p values were compared to indicate significance. All significant variables are shaded in dark gray, with the marginally significant results from Annual Potential Evapotranspiration (PET.A) shaded in light gray.

**Table 3:** Statistics from the principal component analysis and the corresponding phylogenetic ANOVAs of PC1-PC5 on microhabitat use.

Dataset		*PC1*	*PC2*	*PC3*	*PC4*	*PC5*
All Species	Variation	***32*.*77%***	***19*.*76%***	*18*.*89%*	*10*.*70%*	*5*.*60%*
	*Overall Z*	**2.294**	**2.185**	-2.744	0.943	0.109
	*Overall P*	**0.005**	**0.003**	0.992	0.179	0.470
	*Pairwise Z*	**4.644**	**4.198**	-0.547	2.550	1.559
	*Pairwise P*	**0.001**	**0.002**	0.656	0.022	0.088
	*Alpha*	0.010	0.013	0.050	0.017	0.025
Tropical Species	Variation	***45*.*23%***	*24*.*12%*	*10*.*15%*	*6*.*80%*	*3*.*61%*
	*Overall Z*	**1.752**	0.966	-1.972	-2.935	0.121
	*Overall P*	**0.002**	0.129	0.956	0.993	0.547
	*Alpha*	0.010	0.013	0.025	0.050	0.017

PC1 and PC2 show significant differences between arboreal and terrestrial climates (bold) using all species and sequential Bonferroni alpha adjustments under 6-M. The tropical subset analyses (containing only arboreal and terrestrial species) consistently found significant differences between the microhabitat types on PC1. See Supporting information for all Z scores and p values.

**Fig 2:**
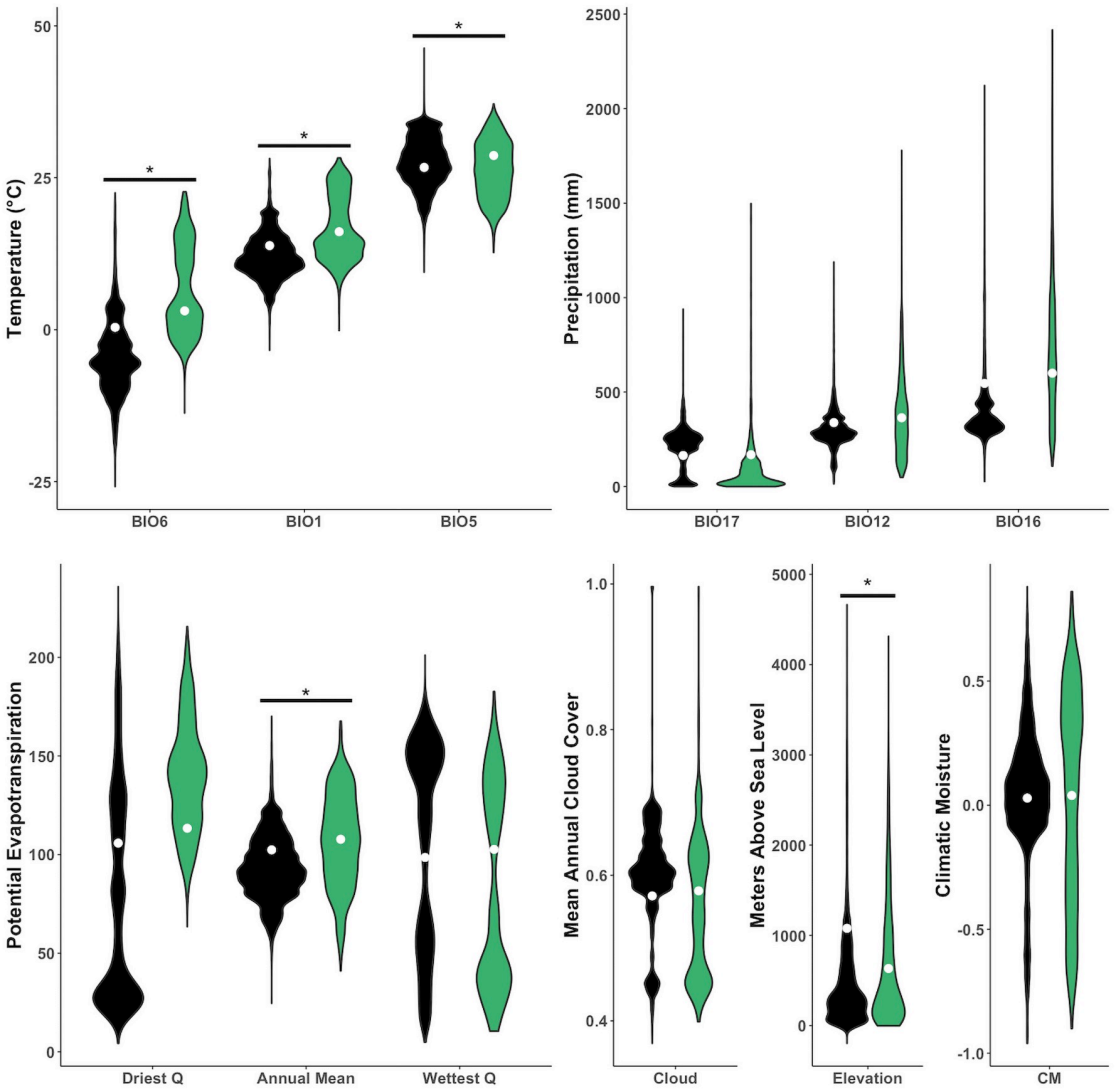
Climatic variables extracted from terrestrial (black) and arboreal (green) species distributions. Specific climatic variables include: Min Temperature of Coldest Month (BIO6), Annual Mean Temperature (BIO1), Max Temperature of Warmest Month (BIO5), Precipitation of Driest Quarter (BIO17), Annual Precipitation (BIO12), Precipitation of Wettest Quarter (BIO16), annual variation in Potential Evapotranspiration (Driest Quarter, Annual Mean, and Wettest Quarter), Cloud Cover, Elevation, and Climatic Moisture. White dots indicate pgls coefficients for each group. Asterisks indicate significant relationships across species means after accounting for phylogeny.

Pairwise comparisons from the individual climatic variable phylogenetic ANOVAs revealed significant differences between arboreal and terrestrial species for all temperature variables (BIO1, BIO5, BIO6; Z > 4.365, p < 0.003) and Elevation (Z_Elev_ = 7.280, p = 0.001; Table 2 and Fig 2) such that arboreal species live, on average, in warmer (Δgls-coefficients: BIO1_mean_ = 2.287; BIO5_mean_ = 1.961; BIO6_mean_ = 2.716), lower elevation (Δgls-coefficients: Elev_mean_ = -444.668) regions than terrestrial species. We also found a marginally significant relationship of Annual Potential Evapotranspiration (PET.A; Z = 3.281, p = 0.010, sequential Bonferroni adjusted α = 0.0063; S3 Table) such that arboreal species live in regions with higher PET.A (Δgls-coefficient of PET.A_mean_ = 64.339).

These patterns were consistent across microhabitat schemes with the exception of the 6-McL classification scheme. With the sequential Bonferroni α adjustments, BIO1, BIO6, and Elevation were only marginally significant for this scheme (p_BIO1_ = 0.025, α_BIO1_ = 0.005, p_BIO6_ = 0.024, α_BIO6_ = 0.0045, p_Elev_ = 0.029, α_Elev_ = 0.0056; S3 Table). Interestingly, this 6-McL analysis revealed a significant difference in the variable Precipitation of the Wettest Quarter (BIO16; Z = 4.173, p = 0.001), although this pattern did not appear elsewhere, even with the tropical 6-McL analysis (S4 Table). Aside from this one exception, we consistently observed arboreal species living in warmer, lower elevation regions with less extreme cold seasons than terrestrial species. Additionally, the 95% confidence intervals of Z scores generated across the 1000 posterior chronograms aligned with these results (S1 Fig), indicating robustness to phylogenetic uncertainty.

For the tropical subset data only including arboreal or terrestrial microhabitat types, each classification scheme determined which species were included in the analyses, thus affecting PC scores. We present the loading schemes from the 6-M tropical analysis, and although the exact weights of each climate variable were slightly different across the classification schemes, the overall patterns were consistent across all tropical schemes. These analyses consistently found differences between arboreal and terrestrial tropical species overall (Z > 2.992, p < 0.003) and across PC1 (Z > 1.670, p < 0.003, S4 Table). The tropical PC1 axis represented 45.23% of the overall variation and was driven by Annual Mean Temperature (BIO1, positive loading), Min Temperature of the Coldest Month (BIO6, positive loading), and Elevation (negative loading; S2 Table).

The tropical analyses of individual climate variables revealed significant differences across all temperature variables and Elevation, as with the main analyses (Z > 2.394, p < 0.003; S4 Table). In addition, these analyses consistently showed variation across all Potential Evapotranspiration variables (PET.A, PET.W, and PET.D) such that tropical arboreal species live in regions with higher PET. Although some of these relationships were only marginally significant under the sequential Bonferroni α adjustment, all PET p-values were less than 0.036 (S4 Table). The 95% confidence intervals generated from the posterior chronograms for the tropical subset also corroborated the findings described above (S2 Fig).

### Ecological niche modeling

**Fig 3:**
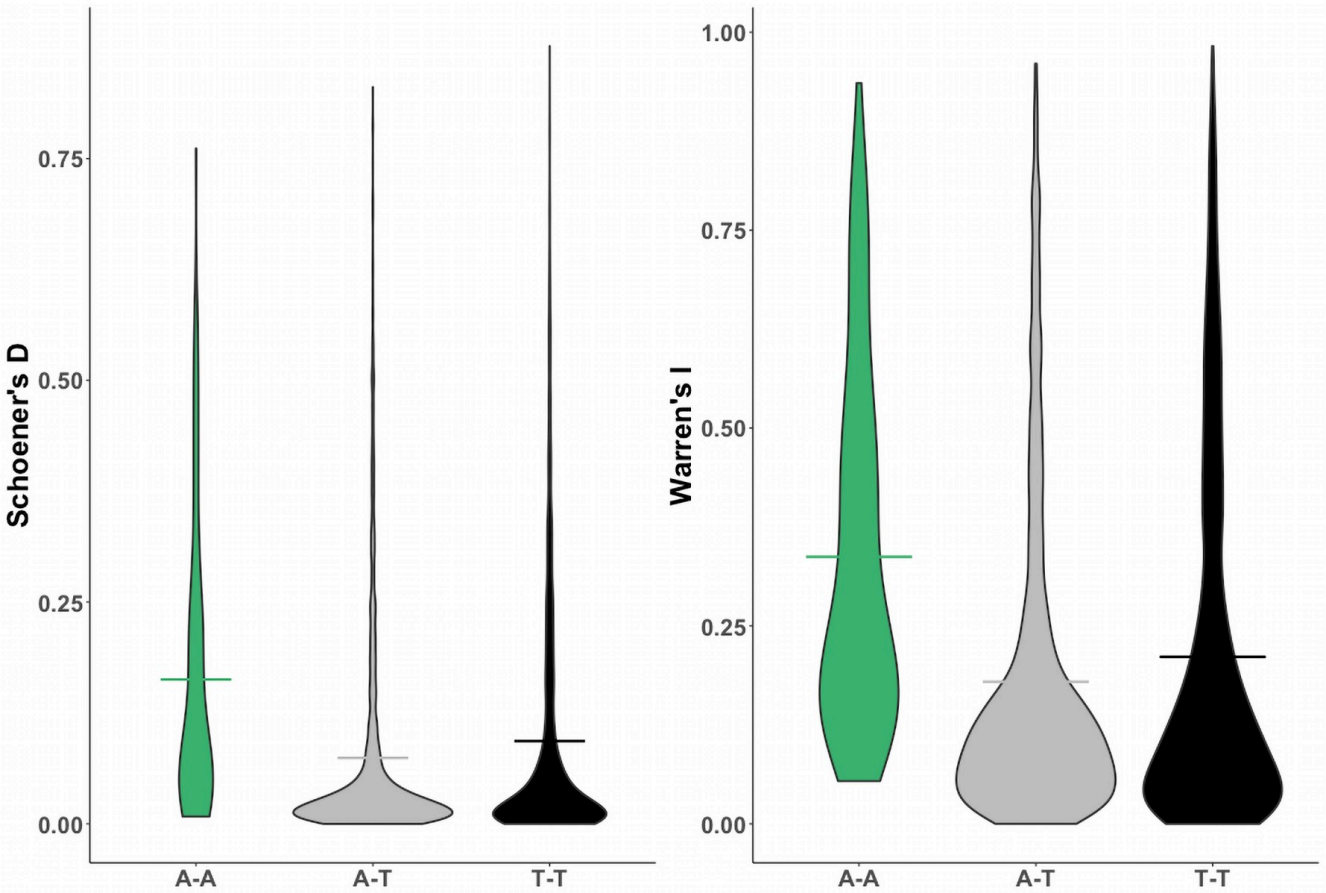
Schoener’s *D* and Warren’s *I* values by comparison type. Horizontal lines indicate group means, all of which differ significantly from one another at an alpha of 0.05. Arboreal species show higher degrees of niche overlap with other arboreal species than with terrestrial species, indicating that arboreality may be constrained by climatic niche.

Species climate models showed high Area Under the Curve (AUC) values from 0.784 to 0.999 (only one model had an AUC less than 0.876), indicating good model performance. Geographic distances ranged from 4.728 to 6961.68 km. As predicted, microhabitat explained variation in the degree of niche overlap for both *D* and *I* values (Z_D_ = 3.179, p_D_ = 0.001, Z_I_ = 3.4443, p_I_ = 0.001). Pairwise comparisons revealed that all groupings were significantly different from each other, such that AT comparisons on average displayed the least amount of niche-overlap, and AA comparisons displayed the greatest amount of niche-overlap (Z > 3.573 p < 0.005; Fig 3).

## Discussion

In many animal clades, microhabitat colonization events are coincident with morphological specialization, indicating that locomotive constraints determine the availability of an unlikely microhabitat (e.g., [53, 54]). However, in salamanders that have repeatedly colonized the arboreal microhabitat, no such morphological explanation exists [4, 5]. In this study, we tested whether broad-scale abiotic conditions differ predictably across ranges of arboreal and terrestrial species to determine whether climate could act as a constraining force in the maintenance of salamander arboreality. Using phylogenetic comparative methods, we tested for correlations between climate and microhabitat type while accounting for phylogenetic relatedness, revealing that arboreal species live in warmer, lower elevation regions than terrestrial species (Table 2 and Fig 2). To complement and expand upon these main results, we employed ecological niche modeling (ENM) by constructing models for each species and calculating niche-overlap between each species pair. The results of this approach were consistent with our main findings, demonstrating that the arboreal and terrestrial species niches are, on average, more divergent from one another than two arboreal species niches, after accounting for geographic and phylogenetic similarity. Terrestrial species pairings were also, on average, more divergent than the other two groupings, indicating that mean climates either differed more greatly among terrestrial species or simply that terrestrial species simply occupy broader geographic ranges. Both possibilities support the PCM findings that terrestrial species are less constrained by climate than arboreal species. Thus, broad-scale climatic patterns may act as a constraint on microhabitat use, influencing salamanders’ life history traits more than previously appreciated.

While our results identify patterns of specific climatic envelopes associated with arboreal species and are generally robust to phylogenetic and microhabitat classification uncertainty, we recognize several important caveats regarding the types of data used in this study. First, as climate data are coarse-grained by nature, data extracted from species occurrence points are likely to capture variation not directly experienced by a salamander. However, while such intraspecific variation in the climatic envelopes experienced by salamanders is ecologically important, we suggest that the fact that we detected significant differences between microhabitat types, in spite of the imprecise nature of these data, warrants consideration, and implies that the patterns are biologically important. Second, our species sampling and location data were limited to the publicly available phylogenetic, environmental, and occurrence data sources. Until more complete phylogenies and occurrence datasets for these excluded species are available, we cannot rectify this uncertainty. We were also unable to include variables such as soil temperature or humidity at the broad scale of this study, as neither measure was publicly available at the necessary scale. Due to the nature of this study, we are only able to report on the correlations observed between microhabitat use and climate. This means that although certain climate variables correlate with microhabitat use, it is difficult to disentangle autocorrelation between certain variables to understand the causal climatic factor in determining microhabitat availability. In other words, although the arboreal salamanders on average experienced warmer temperatures and lower elevation, we cannot conclude that both elevation and temperature are independently necessary for arboreality to evolve or persist. Finally, our findings were based on fairly recent climatic data (since 1970), and therefore, the climate experienced by extant species may not represent what species experienced during the earlier transitions towards arboreality [4]. Similarly, the current geographic localities from which climate data were extracted do not represent the ranges of extinct species that transitioned between microhabitat types. An investigation into ancestral geographic distributions and historical climate would be necessary to define how climate was related to deep historical transitions into and out of the arboreal microhabitat. Overall, the methods used in this study represent the best available approaches for this type of investigation. The consensus across all robustness analyses lend confidence to our conclusions and portends promise for future studies that can expand on this research with improved data resources.

Although our results were largely invariant across phylogenetic and microhabitat classification robustness tests, the 6-McL microhabitat classification scheme did not always yield the same results (loss of signal). These results did show that the arboreal and terrestrial species differ across climate overall (S3 Table), but they did not show the same significant differences across PC2 or BIO5, and differences across PC1, BIO1, and BIO6 were only marginally significant after applying the sequential Bonferroni α adjustment (S3 Table). The major difference in how this classification scheme was defined was the inclusion of species that facultatively climb non-tree vegetation in the arboreal grouping (*sensu* [22]). Classification schemes 6-L and 7-L both included facultatively arboreal species in the arboreal grouping whenever this behavior occurred on trees specifically, but not on other vegetation alone. Thus, the loss of signal with this 6-McL microhabitat classification scheme is likely driven by species that facultatively climb on herbaceous vegetation and other non-tree vegetation, and as such, our results suggest that these species may be able to practice arboreal-like scansorial behavior when micro-climates allow for it without requiring the macro-climate needed by truly arboreal (i.e., tree-dwelling) species. These results suggest that broad climate envelopes may only constrain the use of the arboreal microhabitat, but scansorial behavior utilizing other vegetation may not be restricted in the same manner. Importantly, this same classification scheme did not produce divergent results in the tropical subset analyses, instead showing strong relationships in agreement with other microhabitat classification schemes in the tropics. Thus, the exception of these 6-McL all-species analyses might only apply to temperate species, where we rarely see true arboreality in salamanders. Investigating how facultative scansorial behavior relates to more fine scale weather patterns or species-specific physiological tolerance would likely shed light on this interesting finding.

The tropical analyses were remarkably consistent across microhabitat schemes and corroborated the significant results from the full dataset analyses (S4 Table). We expected that reduction of the data to the tropical region would result in weaker signal, considering the smaller geographic scope inevitably narrows the range of possible climates occupied, as well as the substantial degree of species range overlap between arboreal and terrestrial species in the tropics. Despite this, we observed consistent climatic signal in the tropical clade in agreement with our main results. Additionally, these analyses indicated that tropical arboreal species might also live in regions with higher Potential Evapotranspiration. We found marginally or fully significant results across all relevant measures (PET.A, PET.W, and PET.D) with higher PET averages among arboreal species. Although some of these results were only marginally significant according to the sequential Bonferroni scheme, the consistently low p values (< 0.036) and high Z scores (> 1.623) of these results (plus some marginal PET.A relationships found in the full-dataset; S3 Table) indicate that this pattern might be a valid discrepancy between arboreal and terrestrial species. Potential Evapotranspiration (PET) is the amount of moisture that could be evaporated into the atmosphere, given enough input moisture. Since precipitation (and therefore input moisture) does not differ across microhabitat types, elevated PET values are an indication of higher humidity. The intriguing patterns of higher PET in tropical arboreal species aligns with our prediction regarding the challenges of osmoregulation in arboreal microhabitats: more humid regions would make arboreal microhabitats more hospitable for these animals. However, the PET results presented herein are, at best, interesting relationships that require further investigation.

The consistent results from these analyses demonstrate that arboreal species on average live in warmer, lower elevation regions than terrestrial species. As atmospheric oxygen levels are highest at sea-level and oxygen diffusion rates increases with temperature, results from this manuscript align with the hypothesis that the energetic demands of climbing trees require climates with higher oxygen levels. Thus, our results indicate that climate may constrain arboreality on a macroevolutionary scale due to respiratory challenges, but desiccation threat does not seem to factor into this relationship as we predicted.

This study builds upon the body of work demonstrating how species’ climatic niches have influenced the evolutionary history of plethodontids. Kozak and Wiens [24] first proposed that niche conservatism played a role in driving speciation patterns in North American temperate plethodontids, a hypothesis outlined by Wiens [55]. Soon after, Kozak and Wiens [56] demonstrated that niche conservatism in temperate montane plethodontids promotes speciation as the climate between mountains often creates ecological boundaries between populations, leading to speciation. This work was subsequently expanded upon, showing how family-wide diversification rate shifts often follow climatic-niche evolutionary shifts (e.g., [25, 57–59]. Our work adds another dimension to this story, outlining the possibility that broad-scale climatic patterns determine microhabitat use across the evolutionary history of plethodontids.

Somewhat surprisingly, we discovered that even broad-scale climatic patterns correlate with ecological traits in species with high micro-environment selectivity. A few authors have asserted that small- and large-scale climatic patterns are only weakly associated regarding salamanders [60, but see 12], making the ecological impact of large-scale climate on plethodontids unlikely. However, our study revealed a pattern that is consistent with an ecological response to broad climatic differences, even at the broad resolution of 2.5 arc-minute. This study demonstrated the utility of such coarse climatic data for investigations into Plethodontid ecology and evolution, and we hope these results encourage others to utilize such data despite the widely held assumption that it is not relevant for this group.

This study revealed that arboreal species live in climates that facilitate efficient oxygen uptake, as we predicted was necessary to support arboreal life. These results align with the hypothesis that broad climatic patterns have constrained the availability of the arboreal microhabitat, which could explain why many North American clades have not evolved arboreality despite the abundance of forest habitat. However, the assertion that climate caused deeply rooted microhabitat transitions remains unanswerable with the data at hand; the inconclusive nature of historical biogeographic mapping for plethodontids paired with the challenge of confidently identifying the precise evolutionary time and lineage of microhabitat transitions means that different approaches may be necessary for further investigation into this hypothesis. One such approach could involve monitoring arboreal species in ranges along mountain sides that are predicted to experience rising temperatures under climate change. If suitable temperatures for arboreality become available at higher elevation and arboreal species do not colonize those areas, this could indicate that elevation is constraining arboreality, especially when compared against allopatric terrestrial species. Thus, we present this paper as the groundwork upon which further exploration into the evolutionary relationship between climate and life history traits in Plethodontidae can be built.

## Supporting information

S1 Table Climate summary statistics, number of observations, and microhabitat classifications for each species. Climate summaries include the 5^th^, 25^th^, 75^th^ and 95^th^ percentiles as well as species means and only include data from species with observations from 3 or more climate pixels, as described in the main text. A subset of these species, for which there were 15 or more climate pixels per species, were used for the Ecological Niche Modeling portion of this manuscript. Microhabitat classifications are as follows: arboreal (A), cave (C), fossorial (F), saxicolous (S), terrestrial (T), or aquatic (W).
(XLSX)

S2 Table PCA loadings for all species and tropical arboreal and terrestrial species as defined under 6-M. PC1 and PC2 were significantly different across microhabitat type such that arboreal species displayed higher PC1 values and lower PC2 values for all analyses for which all species were included. Tropical analyses indicated significant differences along PC1, such that arboreal species live in warmer, lower elevation ranges with higher PET of the driest quarter.
(XLSX)

S3 Table General overview of all phylogenetic ANOVA results using all species. Dark gray cells indicate significance against the sequential-Bonferroni adjusted alpha values. Light gray cells indicant significant results at an alpha of 0.05, but only marginal significance against sequential Bonferroni alpha adjustments. All analyses show significance when analyzing all climate variables together. With the exception of microhabitat classification 6-McL, all analyses indicate significant differences across PC1, PC2, all temperature variables (BIO1, BIO5, and BIO6), and Elevation. Annual Potential Evapotranspiration is also marginally or fully significant across all classification schemes except 6-McL. See Discussion for explanation of anomalous 6-McL results.
(XLSX)

S4 Table General overview of all phylogenetic ANOVA results using only tropical arboreal and terrestrial species. Dark gray cells indicate significance against the sequential-Bonferroni adjusted alpha values. Light gray cells indicant significant results at an alpha of 0.05, but only marginal significance against sequential Bonferroni alpha adjustments. All analyses show significance when analyzing all climate variables together, PC1, all temperature variables (BIO1, BIO5, and BIO6), and Elevation. These results all corroborate similar findings when including all species (S3 Table). Potential Evapotranspiration, as quantified by all three PET variables (PET.A, PET.W, and PET.D) are also marginally or fully significant across all classification schemes.
(XLSX)

S1 Fig 95% confidence intervals of the full dataset tests generated across the 1000 posterior chronograms from Bonett and Blair (2017). For all significant patterns described in the main text, the confidence intervals were all above 1.645 (red line), which is the significance cutoff for empirically generated Z scores. This indicates that our results are robust to phylogenetic uncertainty. P values generated from our permutation procedures corroborated these findings.
(PDF)

S2 Fig 95% confidence intervals of the tropical tests generated across the 1000 posterior chronograms from Bonett and Blair (2017). For all significant patterns described in the main text, the confidence intervals were all above 1.645 (red line), which is the significance cutoff for empirically generated Z scores. This indicates that our results are robust to phylogenetic uncertainty. P values generated from our permutation procedures corroborated these findings.
(PDF)
