# Supplementary figures and images for: Is salamander arboreality limited by broad-scale climatic conditions?
Source: PLoS One. 2021 Aug 18;16(8):e0255393. doi: 10.1371/journal.pone.0255393 (PMC8372966; doi:10.1371/journal.pone.0255393)

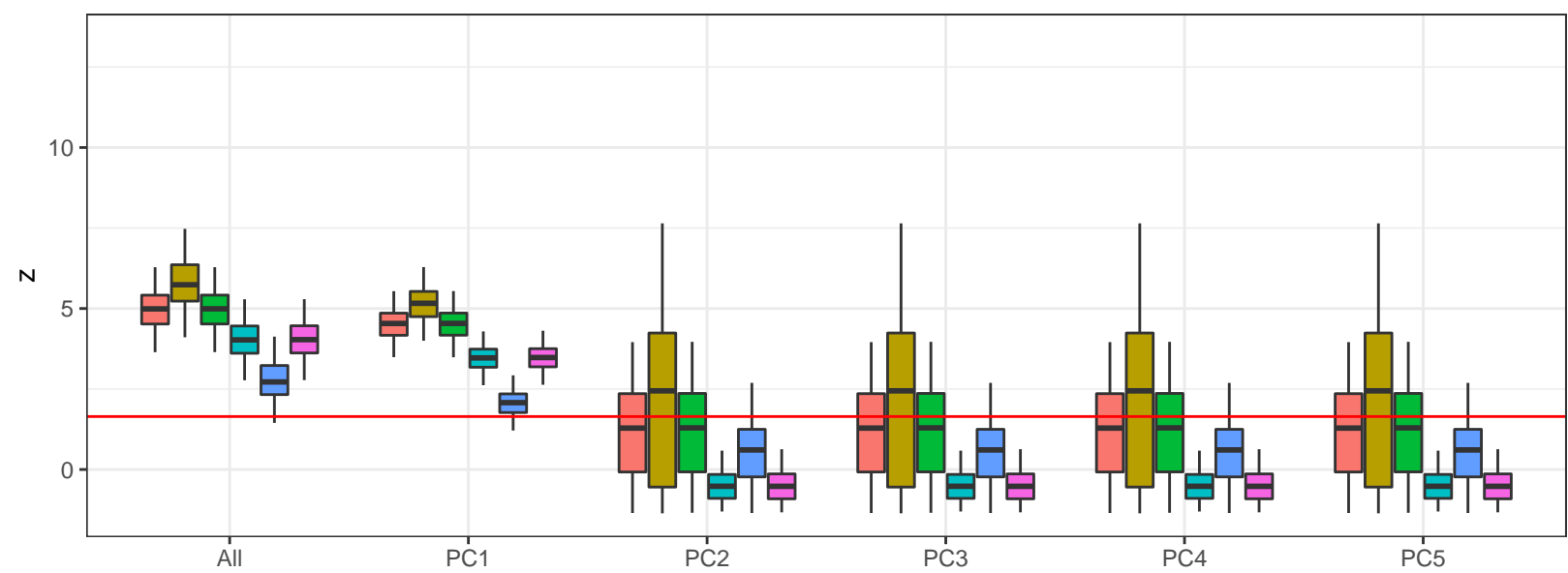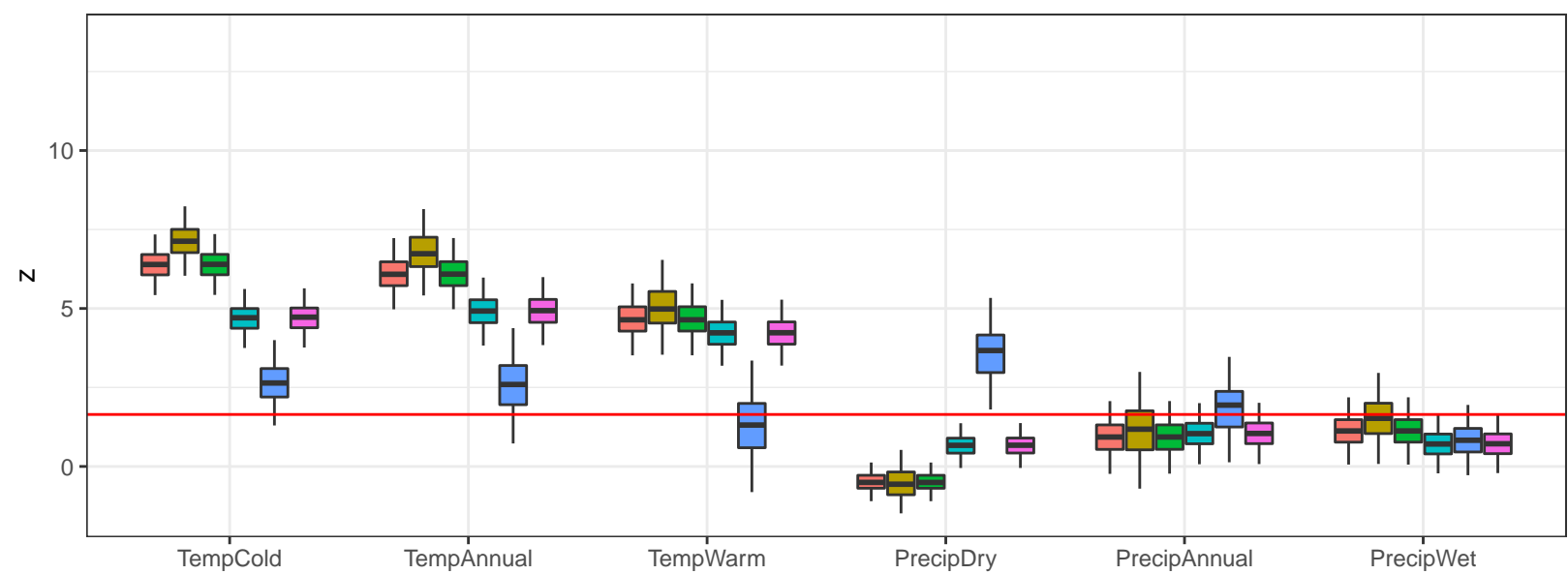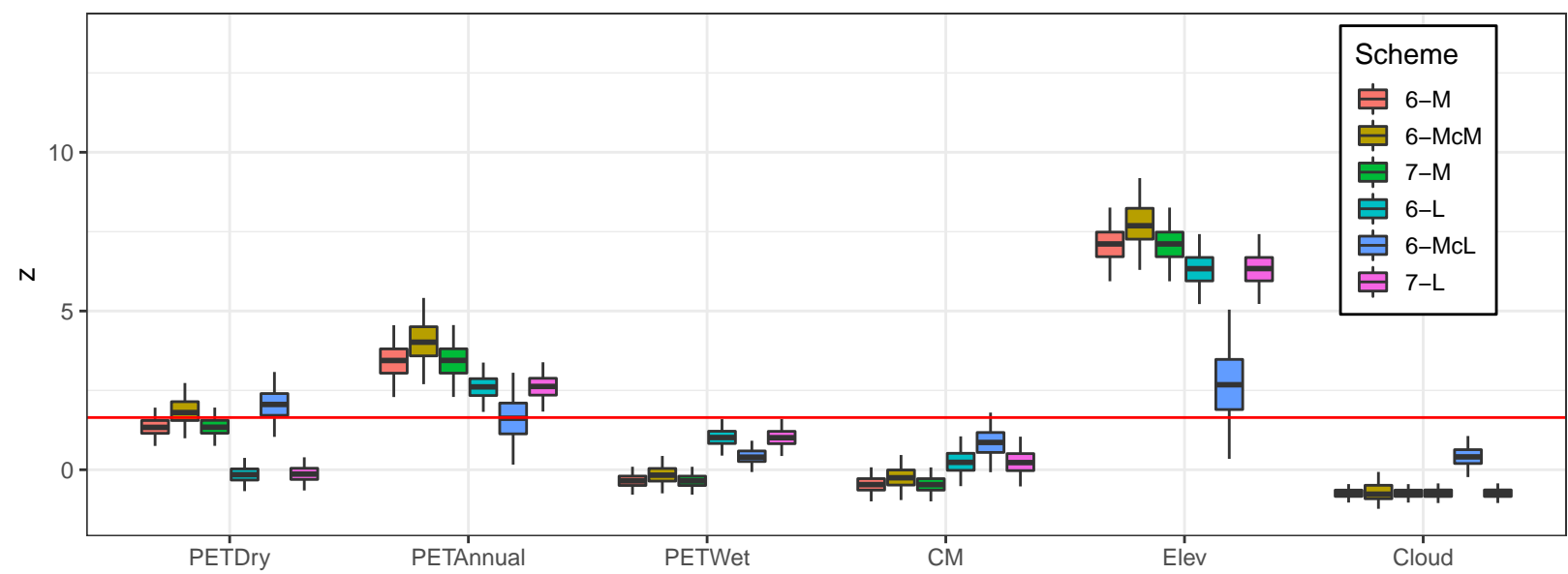

Supplement: S1 Fig — For all significant patterns described in the main text, the confidence intervals were all above 1.645 (red line), which is the significance cutoff for empirically generated Z scores. This indicates that our results are robust to phylogenetic uncertainty. P values generated from our permutation procedures corroborated these findings. (PDF) [file pone.0255393.s005.pdf]

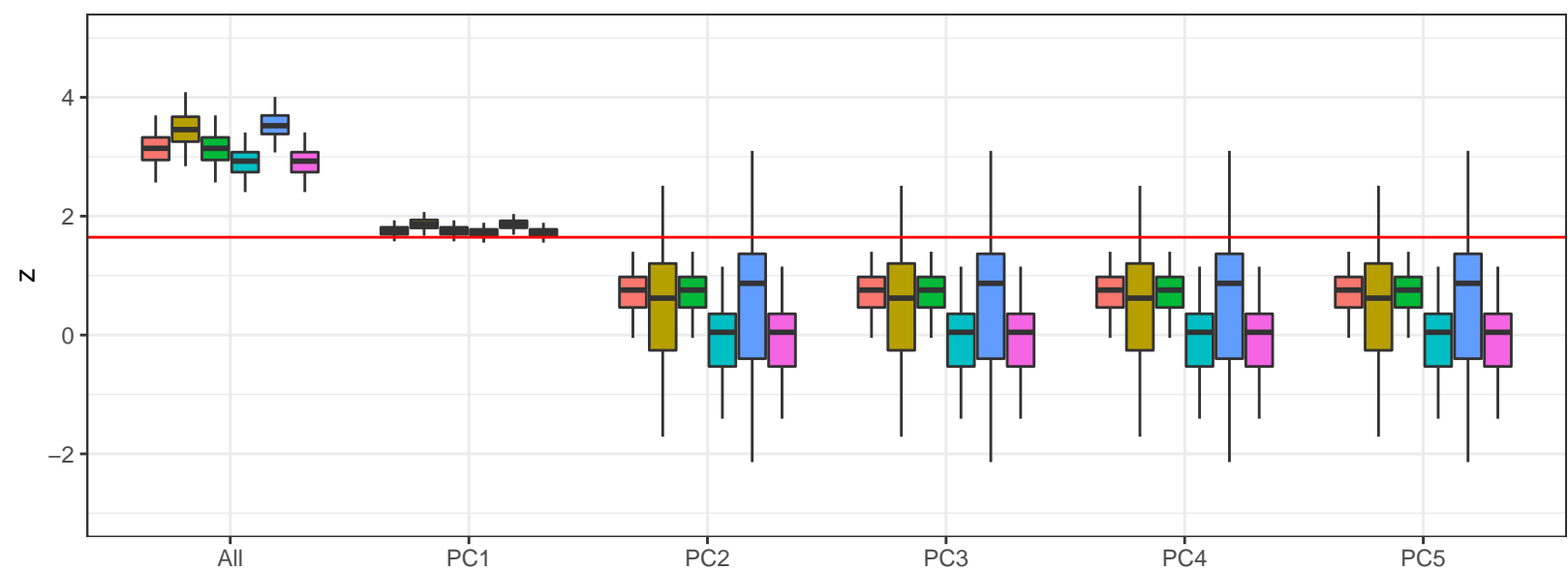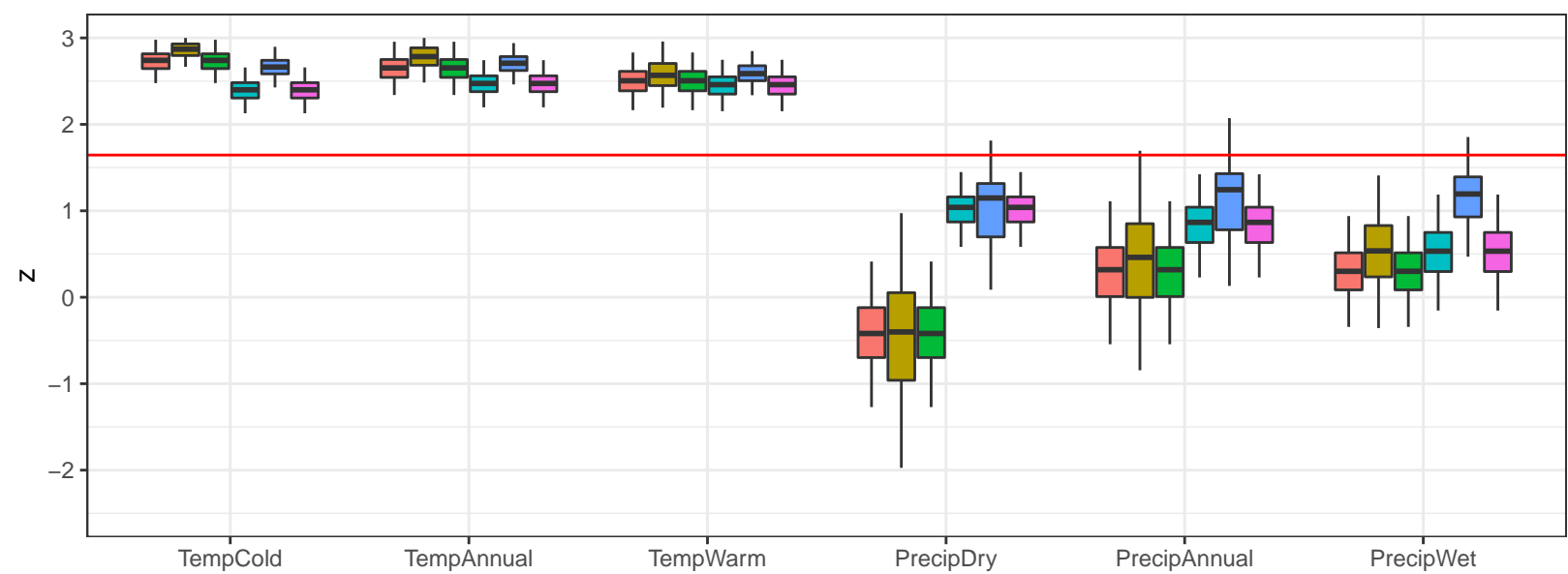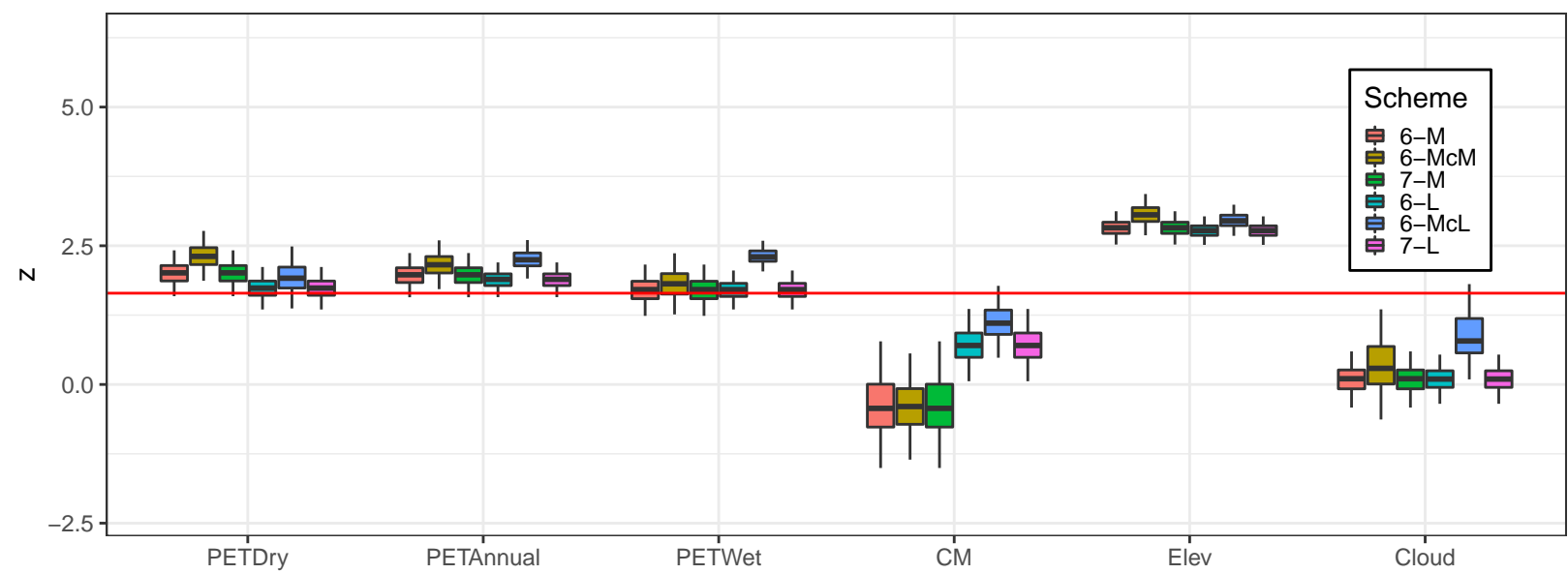

Supplement: S2 Fig — For all significant patterns described in the main text, the confidence intervals were all above 1.645 (red line), which is the significance cutoff for empirically generated Z scores. This indicates that our results are robust to phylogenetic uncertainty. P values generated from our permutation procedures corroborated these findings. (PDF) [file pone.0255393.s006.pdf]
